# Magnetization transfer ratio in the delayed-release dimethyl fumarate DEFINE study

**DOI:** 10.1007/s00415-014-7504-7

**Published:** 2014-10-01

**Authors:** Douglas L. Arnold, Ralf Gold, Ludwig Kappos, Amit Bar-Or, Gavin Giovannoni, Krzysztof Selmaj, Minhua Yang, Ray Zhang, Monica Stephan, Sarah I. Sheikh, Katherine T. Dawson

**Affiliations:** 1 NeuroRx Research, Montreal, QC Canada; 2 Montreal Neurological Institute and Hospital, McGill University, 3801 University Street, Montreal, QC H3A 2B4 Canada; 3 Department of Neurology, St Josef-Hospital/Ruhr-University Bochum, Bochum, Germany; 4 Departments of Neurology and Biomedicine, University Hospital Basel, Basel, Switzerland; 5 Queen Mary University of London, Blizard Institute, Barts and the London School of Medicine and Dentistry, London, UK; 6 Medical University of Lodz, Lodz, Poland; 7 Biogen Idec Inc., Weston, MA USA

**Keywords:** Delayed-release dimethyl fumarate, Magnetic resonance imaging, Magnetization transfer ratio, Multiple sclerosis

## Abstract

We measured changes in brain magnetization transfer ratio (MTR) as a potential indicator of myelin density in brain tissue of patients with relapsing-remitting multiple sclerosis (RRMS) treated with delayed-release dimethyl fumarate (DMF) in the Phase 3 DEFINE study. DEFINE was a randomized, double-blind, placebo-controlled study in which patients with RRMS were randomized 1:1:1 to 2 years of treatment with delayed-release DMF 240 mg twice daily (BID) or three times daily (TID) or placebo. MTR was analyzed in whole brain and normal-appearing brain tissue (NABT) at baseline, week 24, 1 year, and 2 years in a subset of patients. MTR data from 392 patients were analyzed. Mean percentage reduction from baseline to 2 years in median whole brain MTR was −0.386 % in the placebo group vs increases of 0.129 % (*p* = 0.0027) and 0.096 % (*p* = 0.0051) in the delayed-release DMF BID and TID groups, respectively. Similarly, mean percentage reduction from baseline in median NABT MTR was −0.392 % with placebo vs increases of 0.190 % (*p* = 0.0006) and 0.115 % (*p* = 0.0029) with delayed-release DMF BID and TID, respectively. Post hoc analysis of data from patients with no new or enlarging T2 lesions (*n* = 147), or who experienced no relapses (*n* = 238), yielded similar results. In this analysis, increases in MTR in brain tissue most likely reflect increases in myelin density in response to delayed-release DMF. These data in patients with RRMS are consistent with preclinical studies that indicate a potential for cytoprotection and remyelination with delayed-release DMF treatment.

**Electronic supplementary material:**

The online version of this article (doi:10.1007/s00415-014-7504-7) contains supplementary material, which is available to authorized users.

## Introduction

Multiple sclerosis (MS) is a progressive autoimmune disease of the central nervous system, characterized by inflammatory demyelination and neuroaxonal degeneration. In relapsing MS, patients experience episodic relapses associated with neurologic impairment and disability, affecting overall health and quality of life [1]. Relapses are unpredictable, but are understood to be associated with focal inflammation, oxidative stress, and loss of integrity of the blood–brain barrier [2, 3]. Conventional magnetic resonance imaging (MRI) is a sensitive technique for visualizing the focal inflammatory lesions of MS. During relapses, the number of focal lesions detected by MRI increases [4]. Decreases in the number of acute inflammatory lesions in response to treatment are predictive of the treatment effects of disease-modifying therapies on clinical relapses [5].

Although conventional MRI scans are very sensitive to focal white matter pathology in MS patients, diffuse demyelination and axonal degeneration, with consequent neurologic impairment, can progress undetected by standard T1- and T2-weighted MRI imaging techniques [6, 7]. These processes can be detected by non-conventional MRI acquisition techniques [8]. One of these techniques is based on the exchange of magnetization between the pool of protons associated with macromolecules (which are highly concentrated in the membranes of myelin in the brain) and protons associated with water molecules [9]. This phenomenon, which is easily quantified using the magnetization transfer ratio (MTR), can be used to measure and monitor changes in myelin density in the brain over time [10–12].

Changes in the MTR of brain have been shown in animal models to be sensitive to changes in myelin content; MTR decreases with acute demyelination and increases with remyelination [13–15]. Studies performed on post-mortem brains from patients with MS have also shown a strong association between MTR measurements and histopathologically measured myelin content [11, 12]. The MTR of remyelinated lesions differs from both normal-appearing white matter (NAWM) and demyelinated lesions, and there is a significant correlation between myelin content and MTR in both the white matter lesions and the NAWM [12, 16]. Thus, MTR can be used to detect changes in myelin density in normal-appearing brain tissue (NABT) [17] as well as in focal lesions of patients with MS [11, 18, 19] and may prove to be a useful tool for assessing the effects of disease-modifying therapies in MS.

Oral delayed-release dimethyl fumarate (DMF; known as Tecfidera in countries in which it is approved and referred to as BG-12 during clinical development; also known as gastro-resistant DMF) was studied in people with relapsing-remitting MS (RRMS). In two randomized, double-blind, placebo-controlled Phase 3 studies, DEFINE and CONFIRM, delayed-release DMF treatment demonstrated significant clinical and neuroradiologic benefit in patients with RRMS, including significant reductions in the number and volume of MRI lesions relative to placebo [20, 21]. In the DEFINE study, delayed-release DMF BID and TID reduced the mean number of new or enlarging T2 lesions at 2 years by 85 and 74 %, respectively, and the odds of a greater number of gadolinium-enhancing (Gd+) lesions at 2 years by 90 and 73 %, respectively, compared with placebo (all *p* < 0.0001) [21]. The mean number of new non-enhancing T1-hypointense lesions at 2 years was reduced by 72 and 63 % with delayed-release DMF BID and TID, respectively, compared with placebo (both *p* < 0.0001) [Arnold et al. co-submitted to *J Neurol*].

Preclinical studies in tissue and animal model systems demonstrated pleiotropic anti-inflammatory and cytoprotective effects with delayed-release DMF, mediated in part through induction of the nuclear factor (erythroid-derived 2)-like 2 (Nrf2) antioxidant transcriptional pathway [3, 22]. To assess changes in myelin density associated with delayed-release DMF treatment in patients with MS, changes in brain MTR were analyzed in a subset of patients from the DEFINE study.

## Methods

### Study design

Full details of the DEFINE study design, including randomization and blinding, have been reported previously [21]. Briefly, DEFINE was a Phase 3, randomized, double-blind, placebo-controlled, dose-comparison study conducted in 28 countries over a 2-year period in patients with RRMS randomized equally to receive oral delayed-release DMF 240 mg BID, delayed-release DMF 240 mg TID, or placebo. The primary endpoint was the proportion of patients who had experienced an MS relapse at 2 years, assessed in the overall study intention-to-treat (ITT) population (randomized patients who received at least one dose of study treatment). MRI outcomes were assessed in a subset of the ITT population at centers with appropriate imaging facilities (MRI cohort). Secondary MRI endpoints were the number of new or newly enlarging T2 hyperintense lesions and the number of Gd+ lesions at 2 years. Tertiary MRI endpoints included the effect of delayed-release DMF, compared with placebo, on MTR at 1 and 2 years.

The DEFINE study was conducted in accordance with The International Conference on Harmonisation Guidelines on Good Clinical Practice [23] and the ethical principles outlined in the Declaration of Helsinki [24]. Written informed consent was obtained from all patients before evaluations were performed to determine eligibility. Supplemental written informed consent was obtained from all patients who agreed to participate in the MRI portion of the study.

### Patients

Full details of study inclusion/exclusion criteria have been previously reported [21]. Briefly, patients aged 18–55 years with a confirmed diagnosis of RRMS according to McDonald criteria [25] and an Expanded Disability Status Scale (EDSS) score of 0–5.0 were enrolled. Additionally, there had to be documented disease activity, with at least one relapse within 12 months prior to randomization, or a brain MRI scan in the previous 6 weeks showing evidence of at least one Gd+ lesion. Patients were excluded if they had progressive forms of MS, abnormal parameters in pre-specified laboratory tests, other major disease that would otherwise preclude them from participation in a clinical trial, or recent exposure to other contraindicated medications prior to enrollment.

### MRI/MTR methods

Brain MRI scans were performed by blinded MRI technicians at investigational sites whose MRI capability had been validated by the central MRI reading center (NeuroRx Research, Montreal, Quebec, Canada) as described previously [Arnold et al. co-submitted to *J Neurol*]. All original digital data for all MRI images were transferred from each of the sites to the MRI reading center for evaluation by physicians/technicians who were blinded to the patients’ treatment assignments.

MTR analysis was performed in a subset of patients in the MRI cohort as not all MRI sites had the capability to perform MTR assessments. The following MTR data were collected: median MTR of whole brain and NABT at baseline; percentage change from baseline in MTR of whole brain and NABT at week 24, week 48 (year 1), and week 96 (year 2); mean normalized MTR in Gd+ lesion volume (at week 48 [relative to baseline] and week 96 [relative to week 48]); percentage of Gd+ lesion volume with significantly decreased or increased MTR (relative to baseline) at week 48 and 96.

### Quality assurance

MTR data were required to pass the following pre- and post-analysis quality assurance rules:

1. Data from 1T scanners were excluded.
2. Data were excluded from patients with valid scans at only one time point.
3. Data from sites judged unable to provide adequate MTR images were excluded.
4. Scans were reviewed for image quality upon receipt and those scans which failed quality assurance were excluded. Reasons for exclusion included motion artifact and gross image inhomogeneity on the MTR image. If consecutive annual scans showed an extreme change in MTR (more than five standard deviations from the expected mean), the percentage change for this timepoint pair was excluded as this MTR change was considered to be biologically implausible and likely due to technical artifact, e.g., a failing RF amplifier.
5. When a software upgrade occurred, the MTR scans acquired before and after the upgrade were assessed for evidence of a step function change in MTR that affected all tissue types. If such a change was detected, the affected timepoint pair was excluded.

### Statistical analysis

Mean percentage change in MTR (relative to baseline) in whole brain or NABT was compared between treatment groups using analysis of covariance (ANCOVA), adjusted for region and baseline whole brain or NABT MTR value. Data obtained after patients switched to alternative MS medication were excluded. Missing post-baseline data, regardless of reasons, were imputed using mean MTR for each treatment group/visit. Mean normalized MTR in Gd+ lesion volume at week 48 (compared with baseline) and week 96 (compared with baseline) was also analyzed using ANCOVA, adjusted for region. The percentage Gd+ lesion volume with significantly increased or decreased MTR on follow-up scans was compared between treatment groups using the Van Elteren’s test (stratified Wilcoxon Rank Sum test with region as the strata). Post hoc analyses of percentage change from baseline in MTR of whole brain and NABT were performed using data from patients who did not have new or enlarging T2 lesion activity or did not experience a relapse during the study.

## Results

### Patients

The MRI cohort of the DEFINE study consisted of 540 patients, 448 (83 %) of whom had exploratory baseline MTR assessments, and 392 (73 %) of whom had both baseline and post-baseline MTR evaluations. As reported previously, baseline characteristics for the MRI cohort were similar to the non-MRI cohort and the overall DEFINE ITT population and were generally comparable across treatment groups [Arnold et al. co-submitted to *J Neurol*]. For the 448 patients who contributed baseline MTR data, baseline MTR characteristics were similar between treatment groups (Table 1).

**Table 1:** Baseline MTR characteristics

Characteristic	Placebo (*n* = 147)	Delayed-release DMF BID (*n* = 152)	Delayed-release DMF TID (*n* = 149)
Mean (SD) MTR of whole brain	37.1 (5.7)	37.1 (6.1)	37.3 (6.1)
Median (min, max) MTR of whole brain	34.5 (29, 51)	34.0 (28, 51)	36.4 (28, 50)
Mean (SD) MTR of normal-appearing brain tissue	38.1 (5.6)	38.1 (6.0)	38.3 (6.0)
Median (min, max) MTR of normal-appearing brain tissue	35.3 (29, 52)	35.1 (29, 52)	37.5 (28, 51)

### MTR in whole brain and NABT

**Fig. 1:**
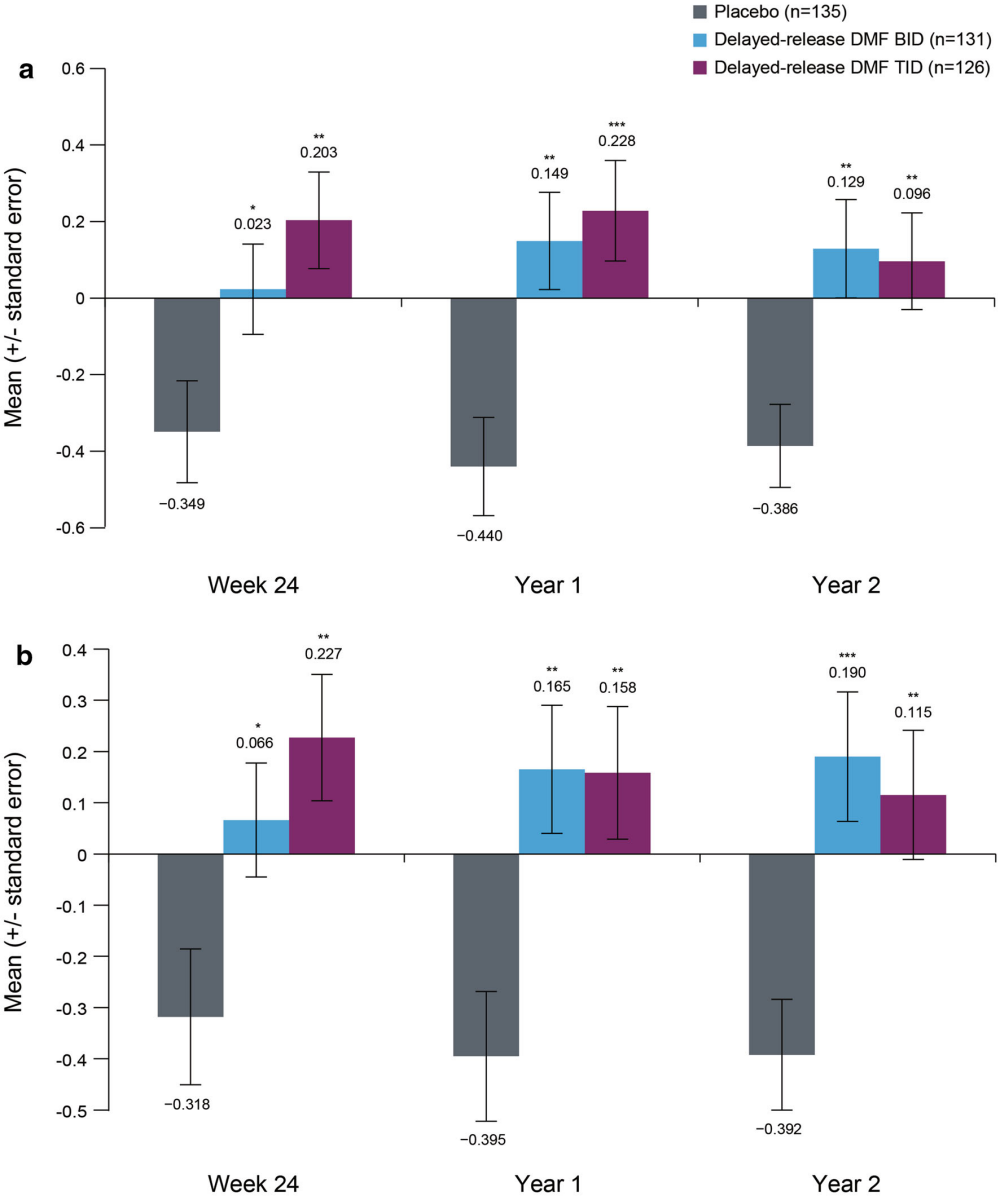
Mean percentage change from baseline in whole brain (**a**) and normal-appearing brain tissue (NABT) (**b**) magnetization transfer ratio (MTR). *p < 0.05, **p < 0.01, ***p < 0.001 vs placebo, based on analysis of covariance, adjusted for region and baseline whole brain or NABT MTR value

After 2 years, there was a mean percentage reduction of 0.386 % from baseline in median whole brain MTR in the placebo group, indicating decreased myelin density. In comparison, there were mean percentage increases of 0.129 and 0.096 % in the delayed-release DMF BID and TID treatment groups, a significant improvement compared with placebo (*p* = 0.0027 and *p* = 0.0051, respectively), suggesting an increase in myelin density (Fig. 1a; Table 2). At 24 weeks and 1 year, increases from baseline in whole brain MTR were observed in both the BID and TID treatment groups that were statistically significant compared with the reductions in MTR that were observed with placebo.

**Table 2:** Mean and median percentage changes from baseline in MTR

	Placebo (*n* = 135)	Delayed-release DMF BID (*n* = 131)	Delayed-release DMF TID (*n* = 126)
Whole brain			
Week 24			
Mean (SD)	−0.349 (1.5455)	0.023 (1.3518)	0.203 (1.4156)
Median (min, max)	−0.320 (−5.03, 3.70)	0.040 (−4.61, 4.94)	0.345 (−4.38, 5.59)
*p* value		0.0481	0.0031
1 year (week 48)			
Mean (SD)	−0.440 (1.4960)	0.149 (1.4519)	0.228 (1.4753)
Median (min, max)	−0.440 (−5.21, 2.94)	0.149 (−3.88, 4.43)	0.228 (−4.36, 4.51)
*p* value		0.0015	0.0003
2 years (week 96)			
Mean (SD)	−0.386 (1.2596)	0.129 (1.4681)	0.096 (1.4151)
Median (min, max)	−0.386 (−4.72, 3.89)	0.129 (−4.47, 4.91)	0.096 (−4.53, 3.58)
*p* value		0.0027	0.0051
Normal-appearing brain tissue			
Week 24			
Mean (SD)	−0.318 (1.5401)	0.066 (1.2718)	0.227 (1.3849)
Median (min, max)	−0.300 (−5.49, 3.44)	0.050 (−4.55, 4.09)	0.227 (−3.44, 5.13)
*p* value		0.0352	0.0027
1 year (week 48)			
Mean (SD)	−0.395 (1.4719)	0.165 (1.4297)	0.158 (1.4540)
Median (min, max)	−0.395 (−4.91, 3.09)	0.140 (−3.73, 4.26)	0.158 (−4.21, 4.67)
*p* value		0.0022	0.0027
2 years (week 96)			
Mean (SD)	−0.392 (1.2582)	0.190 (1.4465)	0.115 (1.4153)
Median (min, max)	−0.392 (−4.29, 3.75)	0.190 (−4.34, 4.73)	0.115 (−4.54, 3.88)
*p* value		0.0006	0.0029

Observed data after patients switched to alternative MS medications are excluded. Missing data prior to alternative MS medications and visits after patients switched to alternative MS medications are included and imputed using the mean of the data for each treatment group/visit. All *p* values were for the comparison between the active and placebo groups, based on analysis of covariance, adjusted for region and baseline whole brain or normal-appearing brain tissue MTR value

Similar findings were obtained for the analysis of median MTR in NABT (whole brain excluding T2-weighted lesions). The mean percentage reduction from baseline to 2 years in median MTR in NABT was 0.392 % in the placebo group, compared with mean percentage increases of 0.190 and 0.115 % in the delayed-release DMF BID and TID groups (*p* = 0.0006 and *p* = 0.0029 vs placebo, respectively) (Fig. 1b; Table 2). At 24 weeks and 1 year, increases from baseline in NABT MTR were observed in the delayed-release DMF treatment groups while reductions in MTR were observed in the placebo group; differences with respect to placebo were statistically significant in both delayed-release DMF groups at both time points.

The robustness of these findings was confirmed by sensitivity analyses using observed data prior to the start of alternative MS treatment, which were consistent with the primary analyses (Supplementary Table 1).

Post hoc analyses of MTR in whole brain and NABT were performed in patients with no T2 lesion activity or those with no relapses during the study. In patients with no new or enlarging T2 hyperintense lesions during the study (*n* = 147), the mean percentage reduction from baseline to 2 years in whole brain MTR was 0.379 % in the placebo group, compared with mean percentage increases of 0.286 % in the delayed-release DMF BID group and 0.170 % in the delayed-release DMF TID group (*p* = 0.0293 and *p* = 0.0538 vs placebo, respectively) (Table 3). Results were similar for NABT MTR in patients with no T2 lesion activity. In patients with no relapses during the study (*n* = 238), the mean percentage reduction from baseline to 2 years in whole brain MTR was 0.347 % in the placebo group, compared with mean percentage increases of 0.232 and 0.096 % in the delayed-release DMF BID and TID groups (*p* = 0.0187 and *p* = 0.0869 vs placebo, respectively) (Table 4). Findings for NABT MTR in patients without relapses were similar. Post hoc analyses at 24 weeks and 1-year, in patients with no T2 lesion activity or those with no relapses, were consistent with the 2-year results, showing reductions from baseline in both whole brain and NABT MTR in the placebo group, compared with either increases or no change from baseline in MTR in the delayed-release DMF groups. The majority of differences relative to placebo were statistically significant in the delayed-release DMF BID and TID groups.

**Table 3:** Mean and median percentage changes from baseline in MTR: patients with no new or enlarging T2 lesions from baseline to 2 years

	Placebo (*n* = 38)	Delayed-release DMF BID (*n* = 58)	Delayed-release DMF TID (*n* = 51)
Whole brain			
Week 24			
Mean (SD)	−0.414 (1.7323)	0.280 (1.3531)	0.401 (1.4342)
Median (min, max)	−0.245 (−5.03, 3.14)	0.145 (−2.15, 4.94)	0.490 (−2.49, 5.59)
*p* value		0.0397	0.0134
1 year (week 48)			
Mean (SD)	−0.506 (1.6290)	0.454 (1.4477)	0.010 (1.2762)
Median (min, max)	−0.415 (−5.21, 1.85)	0.305 (−2.28, 4.43)	0.228 (−4.36, 2.98)
*p* value		0.0019	0.0956
2 years (week 96)			
Mean (SD)	−0.379 (1.5542)	0.286 (1.4307)	0.170 (1.2885)
Median (min, max)	−0.386 (−4.72, 2.37)	0.129 (−2.86, 4.91)	0.150 (−3.92, 2.91)
*p* value		0.0293	0.0538
Normal-appearing brain tissue			
Week 24			
Mean (SD)	−0.299 (1.8041)	0.294 (1.1928)	0.390 (1.3765)
Median (min, max)	−0.015 (−5.49, 3.29)	0.175 (−1.78, 4.09)	0.460 (−2.69. 5.13)
*p* value		0.0561	0.0247
1 year (week 48)			
Mean (SD)	−0.413 (1.6068)	0.434 (1.4000)	−0.017 (1.2119)
Median (min, max)	−0.332 (−4.91, 2.09)	0.165 (−2.45, 4.26)	0.158 (−3.65, 2.46)
*p* value		0.0042	0.1768
2 years (week 96)			
Mean (SD)	−0.312 (1.5918)	0.314 (1.3814)	0.171 (1.2528)
Median (min, max)	−0.392 (−4.29, 2.52)	0.190 (−3.03, 4.73)	0.115 (−3.92, 2.47)
*p* value		0.0285	0.0644

Observed data after patients switched to alternative MS medications are excluded. Missing data prior to alternative MS medications and visits after patients switched to alternative MS medications are included and imputed using the mean of the data for each treatment group/visit. All *p* values were for the comparison between the active and placebo groups, based on analysis of covariance, adjusted for region and baseline whole brain or normal-appearing brain tissue MTR value

**Table 4:** Mean and median percentage changes from baseline in MTR: patients with no relapses from baseline to 2 years

	Placebo (*n* = 62)	Delayed-release DMF BID (*n* = 94)	Delayed-release DMF TID (*n* = 82)
Whole brain			
Week 24			
Mean (SD)	−0.311 (1.7363)	0.002 (1.2743)	0.216 (1.4217)
Median (min, max)	−0.329 (−5.03, 3.70)	−0.040 (−2.82, 4.94)	0.345 (−3.80, 5.59)
*p* value		0.2298	0.0480
1 year (week 48)			
Mean (SD)	−0.589 (1.7264)	0.193 (1.4426)	0.291 (1.5354)
Median (min, max)	−0.440 (−5.21, 2.46)	0.135 (−3.35, 4.40)	0.228 (−4.36, 4.51)
*p* value		0.0028	0.0010
2 years (week 96)			
Mean (SD)	−0.347 (1.5420)	0.232 (1.4074)	0.096 (1.4336)
Median (min, max)	−0.386 (−4.72, 3.89)	0.129 (−2.85, 4.91)	0.096 (−4.53, 3.58)
*p* value		0.0187	0.0869
Normal-appearing brain tissue			
Week 24			
Mean (SD)	−0.323 (1.7329)	0.079 (1.2199)	0.213 (1.3607)
Median (min, max)	−0.254 (−5.49, 3.44)	0.025 (−2.69, 4.09)	0.227 (−3.24, 5.13)
*p* value		0.1054	0.0380
1 year (week 48)			
Mean (SD)	−0.577 (1.6634)	0.194 (1.4615)	0.224 (1.5007)
Median (min, max)	−0.395 (−4.91, 3.09)	0.075 (−3.04, 4.26)	0.158 (−4.21, 4.67)
*p* value		0.0026	0.0023
2 years (week 96)			
Mean (SD)	−0.303 (1.4566)	0.286 (1.3860)	0.096 (1.4318)
Median (min, max)	−0.392 (−4.29, 3.75)	0.190 (−2.84, 4.73)	0.115 (−4.54, 3.88)
*p* value		0.0129	0.1057

Observed data after patients switched to alternative MS medications are excluded. Missing data prior to alternative MS medications and visits after patients switched to alternative MS medications are included and imputed using the mean of the data for each treatment group/visit. All *p* values were for the comparison between the active and placebo groups, based on analysis of covariance, adjusted for region and baseline whole brain or normal-appearing brain tissue MTR value

### MTR in Gd+ lesion volume

The analysis of MTR in Gd+ lesions at 2 years included patients with one or more Gd+ lesions at 1-year. Due to significant suppression of Gd+ lesion activity with delayed-release DMF treatment, data were available for only 7 patients in each delayed-release DMF treatment group compared with 29 in the placebo group. No treatment effect on Gd+ lesion volume MTR endpoints was observed with either delayed-release DMF dose. Mean (median) values of MTR in Gd+ lesion volume were: 0.833 (0.840) in the placebo group, 0.800 (0.830) in the delayed-release DMF BID group (*p* = 0.3922 vs placebo), and 0.781 (0.840) in the delayed-release DMF TID group (*p* = 0.3984 vs placebo). Among the evaluable patients with Gd+ lesions at 1-year, differences were not observed between the placebo group and delayed-release DMF group in the percentage of Gd+ lesion volume that underwent significant increases or decreases in MTR at 2 years.

## Discussion

Delayed-release DMF treatment reduced clinical relapses in patients with RRMS in the Phase 3 DEFINE and CONFIRM studies. Analysis of a cohort of patients from these studies with MRI data demonstrated that delayed-release DMF treatment led to improvements in lesion outcomes compared with placebo in conventional MRI scans [Arnold et al. co-submitted to *J Neurol*]. Improvements in MTR were also observed in both whole brain and NABT in an exploratory analysis of a subset of patients from the MRI cohort who had MTR data acquired.

The use of MTR to assess changes in brain myelin density in response to MS treatments in clinical trials is a relatively new approach that, to our knowledge, has only been used in relatively small scales studies until now [26]. For example, a recent analysis reported a stabilization of grey matter and white matter MTR in 20 patients with RRMS treated with alemtuzumab, compared with a reduction in MTR in 18 untreated patients from a natural history cohort, with a statistically significant difference between groups for grey matter [27]. Our analysis, which uses baseline and post-baseline MTR data from a subset consisting of 392 patients in the delayed-release DMF DEFINE study, represents the largest randomized controlled trial to date to utilize MTR data to study change or stabilization in myelin density in predominantly NABT (whole brain or NABT) in response to MS therapy. Results showed that delayed-release DMF treatment, with either BID or TID dosing, led to significant increases in whole brain MTR and NABT MTR, most likely reflecting increased myelin density. In contrast, patients in the placebo group exhibited a reduction in whole brain and NABT MTR, reflecting the expected decrease in myelin density over time [17]. These findings were evident at 24 weeks and persisted until the end of the study at year 2.

In relapsing forms of MS, remyelination of newly formed lesions can occur in between relapses, so to determine the extent to which normal myelin repair processes may have contributed to the MTR changes observed, we undertook post hoc analyses of MTR data from patients with no new or enlarging T2 hyperintense lesions, and from patients with no relapses, during the 2-year period of the study. Results of these analyses were in accordance with the findings in the overall MTR analysis population: MTR values were reduced among patients receiving placebo, while MTR values in delayed-release DMF-treated patients improved. These data suggest that any remyelination that occurred may be due to a treatment effect of delayed-release DMF on non-lesional tissue rather than due to natural remyelination of lesions.

The analyses of Gd+ lesion volume MTR endpoints were performed to evaluate whether delayed-release DMF had an effect on the evolution of Gd+ lesions that had formed at an earlier time point (1-year). However, due to the suppression of Gd+ lesion development with delayed-release DMF treatment, little data were available and results of these analyses were inconclusive as a result of the small sample size.

A limitation of this study is the interpretation of the extremely small changes in MTR that were measured. The magnitude of these changes is consistent with that reported in another analysis of MTR changes over time, in an untreated cohort [27]. Changes in myelin density of a fraction of a percent can, in principle, be associated with physiological fluctuations such as changes in water content of brain (for example, as a result of inflammation) or decreases in the relative partial volume of cell types other than myelin, for example, axons, astrocytes, or microglia. For these reasons we interpret the observed changes in MTR as reflecting changes in myelin density. Increases in myelin density are consistent with remyelination and decreases are consistent with demyelination, but changes in myelin density of such small magnitude are not specific for demyelination or remyelination.

A previous analysis of MRI data from the DEFINE study has shown that brain atrophy (which reflects axonal loss [28]) was attenuated by delayed-release DMF BID treatment [Arnold et al. co-submitted to *J Neurol*]. Alongside the increases in MTR observed with delayed-release DMF treatment, these findings are consistent with observations from preclinical studies showing a neuroprotective effect of delayed-release DMF in an animal model of experimental autoimmune encephalomyelitis [3, 22, 29–31]. These exploratory analyses support the potential of MTR measurements for detecting treatment effects in large clinical studies of MS therapies; in particular, those thought to have neuroprotective properties that contribute to their mechanism of action.

## Electronic supplementary material

Below is the link to the electronic supplementary material.

Supplementary material 1 (DOCX 27 kb)
