# Supplementary material for: Magnetization transfer ratio in the delayed-release dimethyl fumarate DEFINE study
Source: J Neurol. 2014 Oct 1;261(12):2429–37. doi: 10.1007/s00415-014-7504-7 (PMC4242981; doi:10.1007/s00415-014-7504-7)
Supplement: Supplementary file 1 — Supplementary material 1 (DOCX 27 kb) [file 415_2014_7504_MOESM1_ESM.docx]

# Supplementary Table 1. Sensitivity analyses: mean and median percentage changes from baseline in MTR prior to alternative MS therapy.

|  | **Placebo** | **Delayed-release DMF BID** | **Delayed-release DMF TID** |
| --- | --- | --- | --- |
| **Whole Brain** | | | |
| **Week 24, n** | 131 | 127 | 123 |
| Mean (SD) | –0.349 (1.5691) | 0.023 (1.3731) | 0.203 (1.4329) |
| Median (min, max) | –0.310 (–5.03, 3.70) | 0.060 (–4.61, 4.94) | 0.350 (–4.38, 5.59) |
| *p* value |  | 0.0528 | 0.0039 |
|  | | | |
| **1 Year (Week 48), n** | 117 | 118 | 112 |
| Mean (SD) | –0.440 (1.6079) | 0.149 (1.5304) | 0.228 (1.5656) |
| Median (min, max) | –0.390 (–5.21, 2.94) | 0.045 (–3.88, 4.43) | 0.235 (–4.36, 4.51) |
| *p* value |  | 0.0050 | 0.0017 |
|  |  |  |  |
| **2 Years (Week 96)** | 91 | 108 | 105 |
| Mean (SD) | –0.386 (1.5370) | 0.129 (1.6183) | 0.096 (1.5514) |
| Median (min, max) | –0.410 (–4.72, 3.89) | 0.080 (–4.47, 4.91) | 0.150 (–4.53, 3.58) |
| *p* value |  | 0.0227 | 0.0325 |
| **Normal-Appearing Brain Tissue** | | | |
| **Week 24, n** | 131 | 127 | 123 |
| Mean (SD) | –0.318 (1.5636) | 0.066 (1.2919) | 0.227 (1.4018) |
| Median (min, max) | –0.210 (–5.49, 3.44) | 0.040 (–4.55, 4.09) | 0.230 (–3.44, 5.13) |
| *p* value |  | 0.0399 | 0.0035 |
|  |  |  |  |
| **1 Year (Week 48), n** | 117 | 118 | 112 |
| Mean (SD) | –0.395 (1.5820) | 0.165 (1.5070) | 0.158 (1.5430) |
| Median (min, max) | –0.260 (–4.91, 3.09) | –0.050 (–3.73, 4.26) | 0.160 (–4.21, 4.67) |
| *p* value |  | 0.0070 | 0.0089 |
|  |  |  |  |
| **2 Years (Week 96), n** | 91 | 108 | 105 |
| Mean (SD) | –0.392 (1.5352) | 0.190 (1.5944) | 0.115 (1.5517) |
| Median (min, max) | –0.310 (–4.29, 3.75) | 0.120 (–4.34, 4.73) | 0.100 (–4.54, 3.88) |
| *p* value |  | 0.0093 | 0.0215 |

Observed data after patients switched to alternative MS medications are excluded.

All *p* value s were for the comparison between the active and placebo groups, based on analysis of covariance, adjusted for region and baseline whole brain or normal appearing brain tissue MTR value.
